# Ebola outbreak in DRC and Uganda; an East African public health concern

**DOI:** 10.1002/hsr2.1448

**Published:** 2023-07-31

**Authors:** Sospeter Berling Sospeter, Okereke Promise Udohchukwu, Juvenali Ruaichi, Goodluck Nchasi, Innocent Kitandu Paul, Andrew Marvin Kanyike, Rebecca Susan Dewey

**Affiliations:** ^1^ Weill Bugando School of Medicine Catholic University of Health and Allied Science Mwanza Tanzania; ^2^ Faculty of Dentistry, College of Medicine University of Nigeria Nsukka Enugu Nigeria; ^3^ Department of Internal Medicine Mengo Hospital Kampala Uganda; ^4^ Sir Peter Mansfield Imaging Centre, School of Physics and Astronomy University of Nottingham Nottingham UK

**Keywords:** Democratic Republic of the Congo (DRC), East Africa, Ebola, Ebola outbreak, Uganda

## Abstract

On August 21, 2022, healthcare authorities in the Democratic Republic of the Congo (DRC) announced an outbreak of Ebola virus disease in North Kivu Province, bringing the total to 15 outbreaks nationwide. On September 20, 2022, Uganda's authorities declared an outbreak of the Sudan strain of the Ebola virus following a confirmed a case in Mubende district. As of October 6, 2022, the reported numbers of cases were 63, with 29 deaths in Uganda and 1 case with 1 death in DRC, respectively. Ebola virus causes an acute and severely fatal illness, resulting in death within a very short time if left untreated. In addition, these outbreaks in DRC and Uganda pose a major threat to the health and socioeconomic well‐being of the people of East Africa due to multiple cross‐border activities. Adequate preparations need to be made by the healthcare authorities of the nations concerned; the government, healthcare workers, and the East‐African community as a whole have important roles to play in the effective prevention and control of the spread of Ebola virus within and across their borders.

## Key points

- More than thirty Ebola outbreaks have occurred to date, mostly affecting Western and sub‐Saharan African countries.
- Following recent Ebola outbreaks in Uganda and the Democratic Republic of the Congo, East‐African countries are at a high risk of Ebola outbreak due to high interaction between their citizens.
- Healthcare interventions and efforts employed aimed at stopping previous outbreaks, together with the recommendations presented in this article, are vital for mitigating current Ebola outbreaks and preventing international spread of the virus.

## 1 INTRODUCTION

Eastern Africa, politically known as the East‐African Community, is home to a dynamic population of 470,540,090 people across seven states; Uganda, Tanzania, Kenya, Burundi, Rwanda, Democratic Republic of the Congo (DRC), and South Sudan.^1^ Following official announcements by the World Health Organization (WHO), two East‐African states have recently reported Ebola virus outbreaks. On August 21, 2022, Health authorities in DRC announced an outbreak of Ebola virus disease (EVD) in North Kivu Province.^2^ This outbreak emerged just 4 months after the fourteenth outbreak was declared in the country on April 23, 2022,^3^ bringing the total to fifteen outbreaks. On September 20, 2022, Uganda's health body declared an outbreak of *Sudan ebolavirus* following a confirmed case in Mubende district.^4^ As of October 6, 2022, the reported numbers of cases are 63, with 29 deaths in Uganda, and 1 case and 1 death in DRC.

EVD is caused by the Ebola virus, member of the Filoviridae family and genus Ebolavirus. Three Ebola species; *Zaire ebolavirus* (ZEBOV), *Sudan ebolavirus* (SEBOV), and *Bundibugyo ebolavirus* (BEBOV) have been identified as causing outbreaks in East Africa. The Ebola virus has been detected in blood and other body fluids like saliva, urine, semen, cerebrospinal fluid, tears, and skin swabs.^5^, ^6^ The virus is initially acquired through exposure to the body fluids or tissue of infected animals such as bats and nonhuman primates. Transmission of the disease from the infected animals to humans can occur during hunting and consumption of their food products such as meat. Human‐to‐human transmission occurs through direct contact with body fluids from EVD patients or objects contaminated with infected body fluids. The virus causes an acute and severely fatal illness, resulting in death within a very short time if left untreated. Patients with EVD exhibit sudden‐onset fever, fatigue, muscle pain, headache, and sore throat, followed by vomiting, diarrhoea, kidney and liver damage, and internal and external bleeding.^7^

Ebola outbreaks in DRC and Uganda pose significant threat to the health and socioeconomic well‐being of East‐African people due to frequent international travel in the region. This results in the disruption of daily agricultural activities, trade, and tourism activities, impacting the lives and livelihoods of the people.^8^ Healthcare authorities in the region must be prepared to act.

## 2 EPIDEMIOLOGY OF EVD

**Figure 1:**
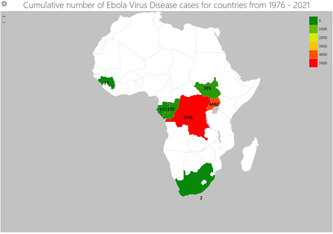
Map of Africa showing Ebola virus cases for different countries between 1976 and 2021, as reported by the Centers for Disease Control and Prevention, 2022.^11^

The earliest cases of Ebola virus infection were reported in Zaire (now DRC) in 1976^9^ Since then, recurrent outbreaks have occurred in Central, Western, and Eastern Africa,^10^ as shown in Figure 1. Multiple EVD outbreaks originated in Central Africa (DRC, Gabon, and the Republic of the Congo) between late 2013 and 2016, when the largest outbreak in history spread from Guinea to other West‐African nations, resulting in 28,652 cases and 11,325 fatalities.^10^ The most recent outbreak between 2018 and 2020 in the Ituri, Nord‐Kivu, and Sud‐Kivu provinces of the DRC was the second largest, with a reported 3478 infections and 2299 deaths (WHO, 2020).

**Figure 2:**
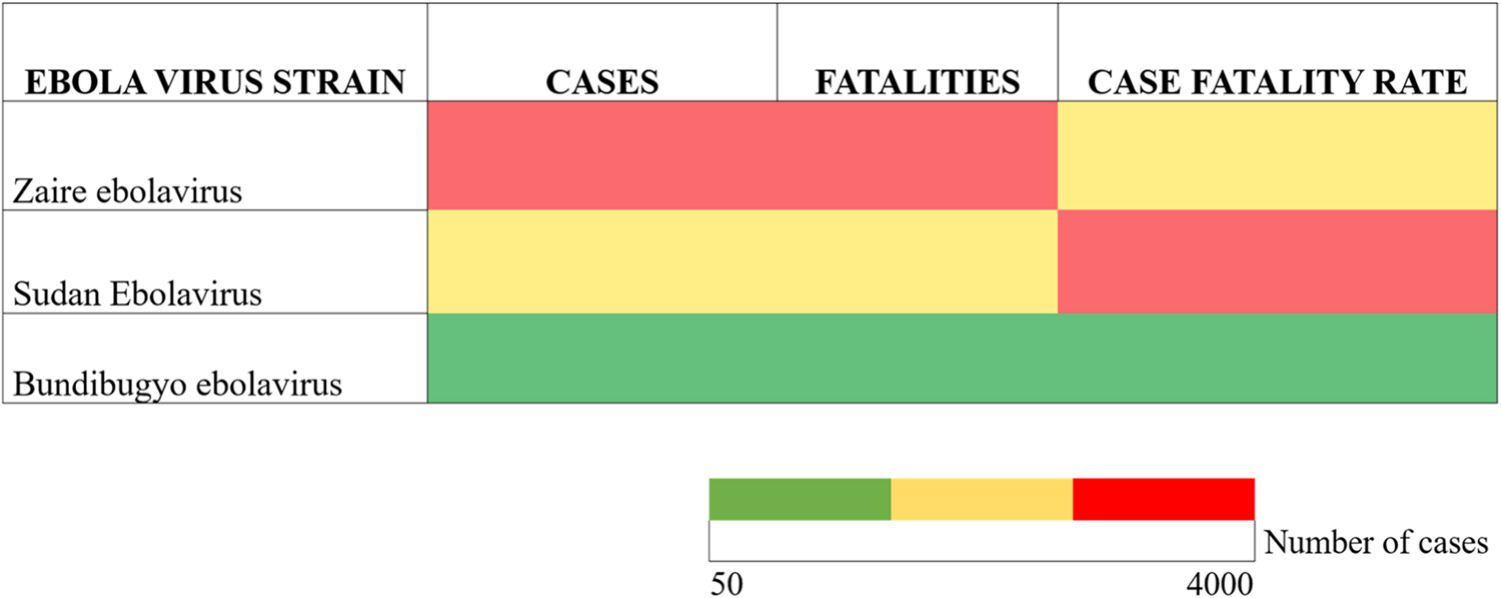
Heat Map corresponding to number of cases and fatalities of different Ebola virus strains for different countries for the period 1976–2021, as reported by the Centers for Disease Control and Prevention, 2022.^11^

The most‐commonly detected species of Ebola virus in EVD outbreaks is ZEBOV, which was associated with the 2014–2016 outbreak in West Africa, and smaller outbreaks in DRC between 2017 and 2021, with a case fatality rate of approximately 90%. Conversely, SEBOV is reported as having a lower case‐fatality rate of between 53% and 65%, with the biggest outbreak occurring in 2000 in Uganda numbering 425 cases. Only one outbreak of BEBOV has been recorded, which occurred in 2007 in Western Uganda; where a case fatality rate of 25% was reported.^12^ Although ZEBOV has caused more outbreaks and cases, SEBOV exhibits a higher fatality rate (Figure 2). The mean case fatality rates (CFR) for each Ebola virus have been estimated at 33.65% (BEBOV), 43.92% (ZEBOV), and 53.72% (SEBOV), giving an overall CFR of between 40% and 50%.^10^

On August 21, 2022, the DRC Ministry of Health announced a new laboratory‐confirmed case of EVD, and 134 hospital contacts were identified. However, no additional confirmed or suspected cases were identified since the official announcement of the outbreak, and on September 27, 2022, the DRC Ministry of Health declared the 15th outbreak in the country to be over.^13^ Nonetheless, this does not rule out the possibility of recurrence as the disease is epidemic in the region.

While this is good news for DRC, Uganda's health authorities are still trying to combat the SEBOV outbreak which tragically affected the districts of Mubende, Kyegegwa, and Kassanda within a very short period. As of September 25, 36 cases were reported with 23 cumulative deaths and 223 contact cases being reported.^14^ The average EVD case fatality rate is approximately 50%, varying from 25% to 90% in previous outbreaks. The incubation period typically ranges from 2 to 21 days. An asymptomatic patient is not believed to be able to spread the disease until they develop symptoms.^15^

## 3 EFFORTS AND RECOMMENDATIONS

### 3.1 Efforts

Health authorities in affected countries have attempted to contain the virus, whereby the countries’ Ministries of Health have strictly imposed health guidelines on citizens attending both public and private facilities, although Uganda ruled out imposing lockdown as it relies on its capacity to contain the virus as it has done before.^16^ Over the past few decades, Uganda has been a relative hotspot of zoonotic disease outbreaks; Ebola Marburg, Yellow fever, and Anthrax. From 2009 to 2014, the country experienced eight outbreaks and three further outbreaks occurred between 2017 and 2018.^17^, ^18^ Despite this, Uganda successfully managed six Ebola virus outbreaks in 2000, 2001, 2007, 2008, 2012, and 2018. These experiences have built strong epidemiological surveillance, preparedness, and response capabilities for Uganda, which it has shared with other countries, including Liberia and Sierra Leone, to help combat the deadliest Ebola outbreak in history (2013–2016). The Ugandan Minister of Health visited the centre of the outbreak, where a meeting was held with the response team and Health Development Partners to discuss the outbreak and capacities to support the response. The need to develop community surveillance and risk communication was highly emphasized.^19^ In collaboration with the WHO and other relevant partners, the DRC Ministry of Health launched response measures to control the outbreak and therefore consolidate the prevention of disease spread.^14^

The WHO has also facilitated investigations, which include identifying the source of contamination, contact‐tracing, boosting the local health capacities at the zone level, organizing outbreak control interventions in the field and include case investigation, strengthening the surveillance system, facilitating isolation of suspected cases and fostering care, laboratory confirmation, and infection prevention and control (IPC) measures in healthcare facilities, in addition to community engagement, social mobilization, defining the risks of exposure in different healthcare departments, and strengthening IPC measures at the hospital.^15^

### 3.2 Recommendations

Controlling the spread of EVD is not only important to DRC and Uganda alone, rather the East African region, Africa and the global community alike. Therefore, the East African region needs to be at alert and be ready to checkmate any route of spread of the virus into its territories.

WHO noted gaps in the DRC's capacity to recover, prepare and respond to outbreaks due to its poor health management systems as contributed to by political unrest and security challenges within the region.^15^ Meanwhile, Uganda on the other hand has been assessed to be experienced in responding to EVD. Nonetheless, concerns arise due to the prevailing circumstances which include unavailability of vaccine to the Sudan strain of the virus, poor contact tracing of the index case and its contacts, poor IPC measures of the hospitals that patients with suboptimal symptoms presented to, burial of Ebola patients with a large gathering, and possible unwillingness of individuals to not adhere to IPC measure should the infection escalate higher. All of these set the overall risk at high national and/or regional level, although low at a global level, cross‐border infections cannot be ruled out even though the district of epicenter has no borders with the international community.^15^, ^20^

We, therefore, recommend the following:

#### 3.2.1 Healthcare/IPC systems’ requirements

Healthcare professionals should immediately report on suspected EVD cases which should include results from laboratory tests. Monitoring of contacts and management of contact tracing activities should be adequately and sufficiently carried out and proper infection prevention measures (e.g., decontamination, safe burials) should be enabled.^20^ Early initiation of supportive treatments should be commenced. There is a need to strengthen surveillance and other response activities to contain the possibility of exponential spread. Furthermore, health workers, contacts, and contacts‐of‐contacts should be vaccinated using the Strategic Advisory Group of Experts (SAGE) recommended vaccine, and for those already vaccinated for more than 6 months should be revaccinated if they are among the contacts or contacts‐of‐contacts of the confirmed case of EVD according to the SAGE. Access to Ebola specific monoclonal antibodies to treat confirmed cases should be provided, as per latest guidelines.^15^, ^20^ Moreover, health professionals should be trained on field testing as vaccination and effective testing and surveillance has been proven to be effective in managing and containing spread.^21^, ^22^ Other vaccines may not be applicable due to their unavailability and rarity of Ebola occurrence globally.^23^

#### 3.2.2 Government's requirements

The EAC governments should maintain their health system adequately to optimal funding and resource‐allocation as these processes keep the systems ready to combat any disease that may occur, rather than being taken up by surprise, which is disastrous to the health system and public health as a whole. Noteworthy, the COVID‐19 pandemic has heavily encroached on the national finance resource envelopes with a worse impact on the health sectors of developing countries that were already underfunded. A multisectoral approach should be employed with the executive governmental arm at the centre to mobilize resources for a quick response. More so, countries should invest in and strengthen their health systems, as this approach strengthens the building blocks, even if there is no imminent risk of an epidemic and reducing the impact should there be an outbreak. Furthermore, countries should conduct surveys to find out the level of acceptability of Ebola vaccines and also set up strategies to improve community participation in EVD control activities.^22^, ^24^

The governments should help in reducing the risk of wildlife‐to‐human transmission through contact with infected fruit bats or monkeys/great apes and consumption of their raw meat by enacting relevant regulations in place to check these practices. The availability of PPE and IPC supplies to manage sick patients and for decontamination should be optimally ensured and provided for by the government.

Engagement with communities to support implementation of preventive behavior and to foster acceptance of outbreak response measures is key and can be achieved through government's interaction and collaboration with the media and community leaders. Moreover, continuous training and retraining of health personnel for early detection, isolation and treatment of EVD cases as well as retraining on safe and dignified burials will go a long way to curtailing cross‐infections between health workers and hospital visitors, and the hospital visitors and those they come in contact with, thus alleviating community transmission.^14^, ^15^, ^21^ Finally, adequate surveillance should be mounted on the borders to checkmate cross‐border infection and also, when necessary, lockdown of the borders and affected districts should be initiated,^14^, ^15^ although the regional adherence to the international health regulations should be the first and main line of action as these regulations curb, prevent and shield against the spread of diseases internationally as well as provide measures for a public health response.^25^

#### 3.2.3 The individuals’ requirements

Animal products (blood, milk, and meat) should be thoroughly cooked before consumption. Direct or close contact with people showing symptoms of Ebola, especially with their bodily fluids should be avoided to reduce the risk of human‐to‐human transmission. Animals should be handled with gloves and other appropriate protective clothing if they are to be touched. Appropriate personal protective equipment should be worn when caring for sick patients and regular hand washing with soap and water is necessary after visiting patients in a hospital, as well as after touching or coming into contact with bodily fluids. WHO recommends that “male EVD survivors practice safer sex for 12 months from the onset of symptoms or until their semen tests negative twice for Ebola virus.” Additionally, individuals should cooperate with the government and healthcare professionals by adhering to IPC guidelines as well as by reporting any case of EVD to the health professionals.^3^, ^14^, ^21^

## 4 CONCLUSION

As Uganda and the Democratic Republic of Congo are striving to limit the spread of the Ebola outbreak, it is crucial to foster both individual and government measures including strict adherence to infection prevention and control guidelines and allocation of enough resources and funds to support the weak healthcare facilities. In addition, public health systems in other East African countries should be ready to contain future outbreaks of Ebola in their regions. Lessons drawn from containment of previous Ebola outbreaks in the region should be employed to respond efficiently to this episode. There should as well be a dialogue to strategize on how to effectively end Ebola outbreaks in this region of Africa.

## AUTHOR CONTRIBUTIONS

**Sospeter Berling Sospeter**: Conceptualization; Project administration; Writing—original draft; Writing—review & editing. **Okereke Promise Udohchukwu**: Writing—original draft; Writing—review & editing. **Juvenali Ruaichi**: Writing—original draft; Writing—review & editing. **Goodluck Nchasi**: Writing—original draft; Writing—review & editing. **Innocent Kitandu Paul**: Writing—review & editing. **Andrew Marvin Kanyike**: Writing—review & editing. **Rebecca Susan Dewey**: Writing—review & editing.

## CONFLICT OF INTEREST STATEMENT

The authors declare no conflicts of interest.

## TRANSPARENCY STATEMENT

The lead author Sospeter Berling Sospeter affirms that this manuscript is an honest, accurate, and transparent account of the study being reported; that no important aspects of the study have been omitted; and that any discrepancies from the study as planned (and, if relevant, registered) have been explained.

## Data Availability

Data sharing not applicable as no new data were generated or analysed.
